# Alpha Amylase as a Salivary Biomarker of Acute Stress of Venepuncture from Periodic Medical Examinations

**DOI:** 10.3389/fpubh.2014.00121

**Published:** 2014-08-26

**Authors:** David Koh, Vivian Ng, Lin Naing

**Affiliations:** ^1^PAPRSB Institute of Health Sciences, Universiti Brunei Darussalam, Gadong, Brunei Darussalam; ^2^SSH School of Public Health, National University of Singapore, Singapore, Singapore

**Keywords:** salivary biomarker, alpha amylase, venepuncture, acute stress, periodic medical examination

## Abstract

Periodic occupational health examinations often require venepuncture. Acute psychological and physical stressors during such procedure result in sympathetic stimulation and increased salivary protein secretion, including salivary α-amylase (SAA). We studied SAA response to venepuncture during such examination. Fifty-eight healthy males undergoing periodic medical examination reported perceived stress level (PSL) scores (on a five-point scale) and provided passive drool saliva samples at 15-min (T1) and 1-min before (T2); and 1-min (T3) and 15-min after venepuncture (T4). A subset of 33 participants available for repeat examination on a control day when there was no venepuncture provided saliva samples at the corresponding times for comparison. Saliva SAA activity levels were analyzed using a SAA assay kit (Salimetrics LLC, USA). Among 58 participants, mean SAA increased from T1 (89.95 U/L) to T2 (109.5 U/L) and T3 (116.9 U/L). SAA remained elevated 15 min after venepuncture (121.0 U/L). A positive trend in the difference of SAA between T3 and T1 was noted among subjects with increasing mean PSL scores. T3–T1 values were 0.6 (among those with PSL ≤ 1, *n* = 24), 11.3 (among those with PSL between 1 and 1.5, *n* = 18), and 78.9 (among those with PSL > 1.5, *n* = 16). SAA increment over four-time points was significantly higher on the venepuncture compared to the control day (*P* = 0.021). SAA increases in response to the acute stress of venepuncture during a periodic medical examination, and remains elevated 15 min after the procedure. In comparison, such fluctuations in SAA were not seen on a control day. During venepuncture, increase in SAA from baseline is higher among those who reported greater self-perceived stress during the procedure.

## Introduction

The use of periodic medical examinations for occupational health surveillance of workers exposed to workplace hazards is a well-established practice. Such examinations may be a simple review of symptoms associated with exposure to a specific hazard, but often require measurement of physiological parameters or biological monitoring of biomarkers of exposure and effect (1). The extraction of venous blood for biological monitoring is a common procedure for workers. This procedure will cause psychological stress and pain for most the people regardless of age (2, 3).

Previous studies have indicated that salivary α-amylase (SAA) levels increase acutely in response to physical (4–6) as well as psychological stressors (7–12). Most of the studies to assess psychological stress were carried out in a laboratory or in quasi-naturalistic settings (9). It was suggested that the secretion of SAA responds to stimulation of the sympathetico-adreno-medullary (SAM) system. This is based on the fact that sympathetic stimulation increases salivary protein secretion (10). Salivary flow rate has also been shown to have negligible impact on stress-induced SAA activity (13). Another finding is that SAA levels can also increase in response to pain. In a study conducted by Shirasaki et al. to investigate the correlation between SAA activity with pain intensity, a lumbar epidural block significantly decreased SAA activity in patients with chronic pain (14).

In this study, we examined the acute stress (using SAA as a marker) among workers who were subjected to a periodic medical examination, which required a venepuncture to be performed. We also investigated the association between changes in SAA and subjects’ self-reported stress levels during the procedure.

## Materials and Methods

### Participants

The study was conducted in conjunction with the routine six-monthly statutory medical examinations of a group of workers employed in a factory manufacturing a lead (Pb) based polyvinyl chloride stabilizer located in the western part of Singapore.

Periodic medical examinations of lead exposed workers by a registered designated workplace doctor are required by law in Singapore. The medical examination involves sampling of venous blood for measurement of hemoglobin and lead.

Approval to conduct this study was obtained from the NUS Institutional Review Board (NUS IRB reference code 05-045).

All 58 male workers working in the factory who were exposed to lead were examined during the statutory periodic medical examination over two working days. Two weeks later, we collected the saliva samples on a control day (at the same time of day) from a subset of 33 workers from the originally studied group of 58 workers. These 33 workers were selected on the basis of their availability at the workplace on the control day. During the control day, the 33 participants were gathered in a room and occupied with reading newspapers/magazines.

All workers were briefed about the study and gave written informed consent before participation. Inclusion criteria of participants for the study were males who did not suffer from any occupational or non-communicable non-occupational diseases, including pre-existing psychiatric illness. Workers were advised not to smoke, eat, or consume caffeinated drinks 1 h prior to saliva collection.

### Saliva sampling and analysis

Saliva samples were collected from each subject at 15-min (T1) and 1-min (T2) before venepuncture/control day activities, and 1-min (T3) and 15-min (T4) after venepuncture/control day activities. At the same time points, subjects were asked to rate their perceived stress levels (PSLs) on a 5-point scale (ranging from 1 = not stressed at all, to 5 = extremely stressed) prior to saliva collection. Participants were required to spit through a straw into a microcentrifuge tube to obtain a volume of approximately 1 mL at each time point.

Saliva collection was performed by research personnel who were trained in saliva collection techniques.

Salivary α-amylase activity (U/L) in each sample was measured using a kinetic measurement kit (SAA assay kit, Salimetrics LLC, USA).

### Statistical analysis

Descriptive statistics were used to describe demographic data and SAA levels. Repeated measures ANOVA (RMANOVA) was used to compare four-time points measures of SAA and PSL among 58 participants. RMANOVA was also used to compare the pattern (through four-time points, T1–T4) of SAA levels between venepuncture and control days among the 33 participants. Required assumptions of RMANOVA such as normality, sphericity, and equal-variance, were checked. As the normality and sphericity assumptions were not met in all RMANOVA analyses, log-transformed SAA and PSL data, and the Greenhouse–Geisser correction procedure were used, respectively. Although there was a wide variation of age in the study sample, inclusion of age as a covariate in the RMANOVA model did not change the result and therefore, it was not included in the final model.

Data were analyzed with IBM SPSS Version 20.0 (SPSS Institute, Chicago, IL, USA). All hypothesis tests were two-sided and *P* values <0.05 were considered as statistically significant.

## Results

### Demographic data

All male 58 lead exposed workers who worked in the factory participated in the study. Their ages ranged between 22 and 61 years, with a mean (SD) age of 36.1 (10.7) years. Their duration of employment at this particular workplace ranged from few months to 24 years. The blood lead levels of all the workers were below 50 μg/dL.

A subset of 33 male workers [mean (SD) age 36.4 (9.7) years] who were available on a control day 2 weeks later were re-examined while they were reading newspapers.

### Change in perceived stress level and salivary α-amylase of 58 workers

The 58 subjects who underwent the periodic medical examination indicated that venepuncture was not a stressful procedure, with a PSL score of 1 (76% at T1 to 83% at T4). More subjects (40% at T2 to 47% at T3) indicated they were more stressed (PSL of 2 or more) immediately before and after the venepuncture.

**Figure 1:**
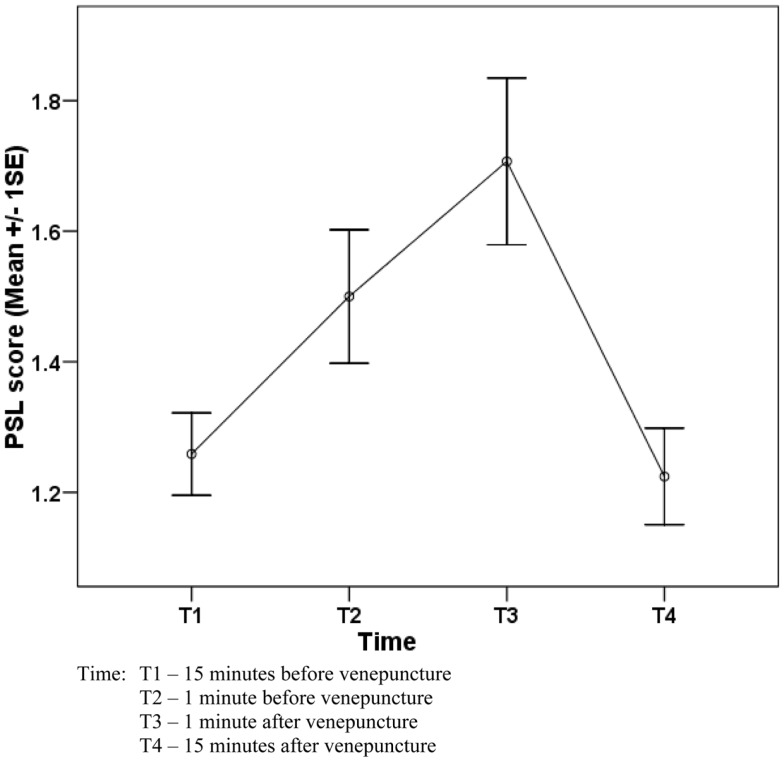
**Change in perceived stress level (PSL) of 58 workers before and after venepuncture**.

**Figure 2:**
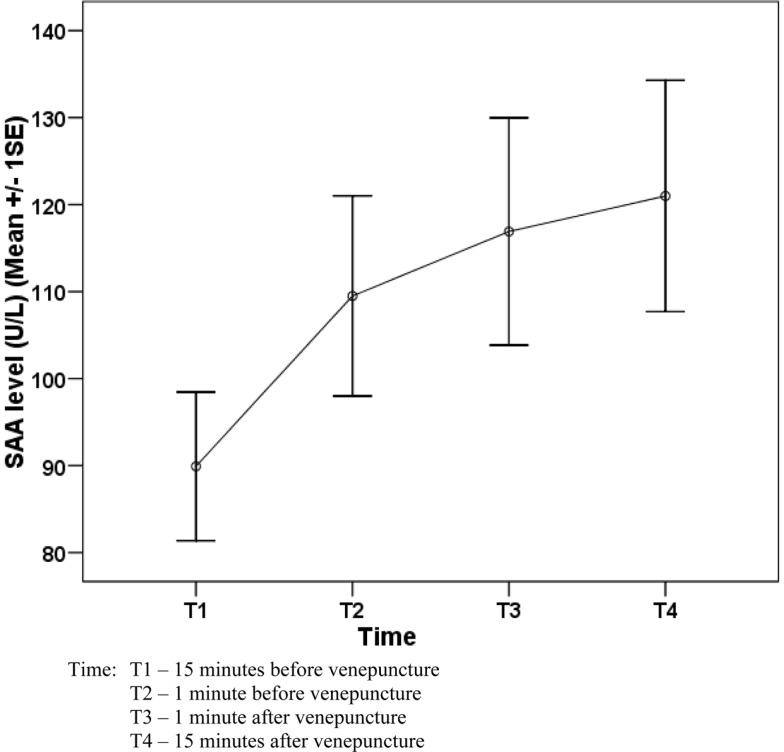
**Change in salivary α-amylase (SAA) activity level (U/L) of 58 workers before and after venepuncture**.

The pattern of the mean scores of PSL and mean SAA levels over the four-time points is presented in Figures 1 and 2.

Table 1 shows the SAA activities in four-time points among the 58 subjects who underwent the periodic medical examination and the venepuncture. Overall, there was a significant change of SAA level (*P* = 0.001) and the significant change was detected between T1 and T2 (*P* = 0.039). As there was a variation of age in the sample, age variable was added into the model to control the effect of age. However, there was no significant change in the result.

**Table 1:** **Change of salivary α-amylase (SAA) level at four-time measures of 58 workers before and after the venepuncture**.

Time	Salivary α-amylase (U/L)		*P* value
	Mean	SD	
T1
15 min before venepuncture	89.9	65.1	0.002 (Overall)^a^
T2
1 min before venepuncture	109.5	87.7	0.036 (T1 vs. T2)^b^
T3
1 min after venepuncture	116.9	99.4	0.861 (T2 vs. T3)^b^
T4
15 min after venepuncture	121.0	101.3	1.000 (T3 vs. T4)^b^

*^a^Repeated measures ANOVA for four-time measures with log transformation (with Greenhouse–Geisser’s epsilon correction) (*F* = 6.59; df = 3;130)*.

*^b^*Post hoc* three multiple comparisons with Bonferroni’s procedure*.

Table 2 shows the PSL score at four-time points among the 58 subjects who underwent the periodic medical examination and the venepuncture. Overall, there was a significant change of PSL score (*P* < 0.001) and the significant change was detected between T1 and T2 (*P* = 0.018) and T3 and T4 (*P* < 0.001).

**Table 2:** **Change of perceived stress level (PSL) at four-time measures of 58 workers**.

Time	Perceived stress level		*P* value
	Mean	SD	
T1
15 min before venepuncture	1.3	0.48	<0.001 (Overall)^a^
T2
1 min before venepuncture	1.5	0.78	0.054 (T1 vs. T2)^b^
T3
1 min after venepuncture	1.7	0.97	0.051 (T2 vs. T3)^b^
T4
15 min after venepuncture	1.2	0.56	<0.001 (T3 vs. T4)^b^

*^a^Repeated measures ANOVA for four-time measures with log transformation (with Greenhouse–Geisser’s epsilon correction) (*F* = 10.63; df = 3;115)*.

*^b^*Post hoc* three multiple comparisons with Bonferroni’s procedure*.

Although PSL score significantly dropped from T3 to T4, a slight increase of SAA was observed from T3 to T4 (though it was statistically not significant).

A positive trend in the difference of the SAA level between T3 and T1 was noted among subjects categorized by increasing mean PSL scores (Table 3).

**Table 3:** **Change in SAA (T3–T1) among participants categorized by mean PSL score**.

Mean PSL score (T1–T4)	*n*	SAA change^a^ (T3–T1) (U/L)			
		Mean	(SD)	Median	(IQR)
≤1.00	24	0.6	41.2	1.6	59.5
1.01–1.50	18	11.3	50.4	1.8	32.2
1.51+	16	78.9	113.2	29.5	179.1
Total	58	27.0	75.7	5.3	57.1

*^a^Minimum and maximum values were −69 and 307, respectively*.

### Comparison of salivary α-amylase levels of the 33 workers during the venepuncture and the control day

All SAA levels of the 33 workers were positively skewed, which was shown by mean and standard deviation (SD) in Table 4. Due to the skewness of residuals in using RMANOVA in comparing two patterns (venepuncture and control days), log-transformed data were used for the comparison.

**Table 4:** **Comparison of pattern of salivary α-amylase (SAA) of 33 workers on venepuncture and control days**.

Time	SAA level (U/L)				RMANOVA^a^
	Venepuncture		Control day		*F* Stat, (df), [*P* value]
	Mean	SD	Mean	SD	
T1	75.5	51.73	95.9	56.68	5.92, (1; 32), [0.021]^b^
T2	92.1	72.47	99.4	65.02	3.23, (1; 32), [0.082]^c^
T3	94.0	81.00	94.2	66.49	1.15, (1; 32), [0.291]^d^
T4	97.2	77.03	98.6	66.34	0.43, (1; 32), [0.516]^e^

*^a^Repeated measures ANOVA using log-transformed SAA data*.

*^b^Comparing pattern (T1, T4)*.

*^c^Comparing pattern (T1, T2)*.

*^d^Comparing pattern (T2, T3)*.

*^e^Comparing pattern (T3, T4)*.

*T1, 15 min before venepuncture or control day reading activity*.

*T2, 1 min before venepuncture or control day reading activity*.

*T3, 1 min after venepuncture or control day reading activity*.

*T4, 15 min after venepuncture or control day reading activity*.

Comparison of the pattern of log-transformed SAA levels between venepuncture and control days is presented in Table 3.

**Figure 3:**
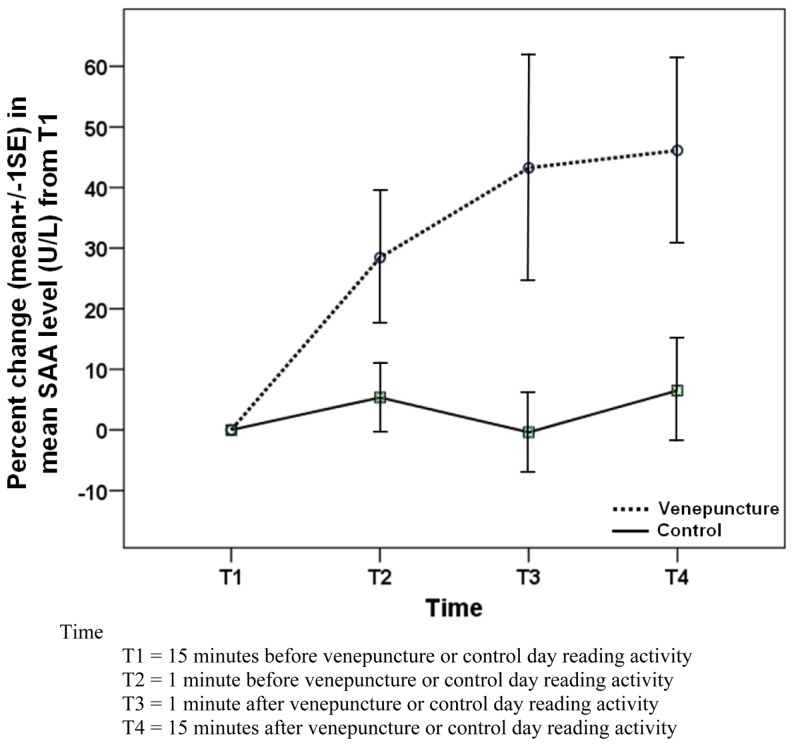
**Comparison of change in SAA level between venepuncture and control days among 33 subjects**.

The comparison of pattern (T1–T4) was statistically significant (*P* = 0.021) although all the rest in between patterns (T1–T2), (T2–T3), and (T3–T4) were statistically not significant between venepuncture and control days (Figure 3). As there was a wide variation of age in the sample, age variable was added into the model to control the effect of age. However, there was no significant change in the result.

In view of the statistically negative results, a power calculation was done for the sample size of 33. To detect the difference of 20 units of SAA level (if we consider that 20 units difference of change in SAA between two study groups is an important finding to detect), the power of the study was 26.2%, for an alpha of 0.05. To achieve a study with 80% power, we would require 142 participants. The within-group largest SD (T1–T4) of SAA was 60 U/L, estimated from the data for the power calculation.

## Discussion

Amylase is a calcium-containing metalloenzyme, which has a number of isoenzymes. In saliva, it is produced by serous acinar cells of the parotid and submandibular salivary glands, and accounts for 10–20% of total salivary proteins. The parotid glands produce 80% of all SAA. An important function of SAA is to hydrolyze α-1,4 linkages of starch to glucose and maltose. This property allows SAA quantification by measuring its activity in enzyme units per milliliter, where an enzyme unit is the amount of enzyme that catalyzes conversion of 1 μmol of substrate per minute. SAA also contributes to oral cavity mucosal immunity by inhibiting adherence and growth of bacteria.

Salivary glands are innervated by both sympathetic and parasympathetic nerves, and increase in SAA is a reflection of autonomic nervous system activity. Sympathetic nerve stimulation via norepinephrine increases total salivary protein concentration, while parasympathetic cholinergic stimulation increases salivary flow rate. As such, SAA activity increases with physical stressors, pain, and psychological stress. Most of the research on SAA took place in experimental laboratories or quasi-naturalistic settings, but field studies e.g., of acute anticipatory stress and pain due to venepuncture, also show increase and continued elevation of SAA.

Among 58 participants, SAA increased from T1 (mean 89.95 U/L) to T2 (109.5 U/L) and T3 (116.9 U/L). SAA remained elevated 15 min after venepuncture (121.0 U/L). The results indicate a significant elevation of SAA from 15 min before venepuncture to 1 min before venepuncture. For many people, venepuncture is a painful procedure, which can induce certain level of psychological stress, and it is not surprising to note that the levels of SAA begin to increase even before the subjects underwent venepuncture. This may be due to the stress of anticipation of the painful procedure. It has been reported the psychological stress activates the SAM system (4, 10) and results in increased SAA release.

The means SAA levels remained elevated 15 min after the venepuncture. In comparison, a study of subjects’ response to the “Trier Social Stress Test (TSST)” (a psychosocial stress test), showed that SAA levels decreased to the control session level after 20 min (11). Another study conducted by Gordis and colleagues on adolescents’ response to TSST, showed saliva α-amylase levels returning to baseline 10 min post-stressor (7).

Our findings may be due to the study subjects still experiencing residual pain after the venepuncture. Continuing painful stimuli may also activate the SAM system (15, 16). Shirasaki et al. (14) suggested that SAA activity may be a good index for the objective assessment of pain intensity and may reflect activity of the SAM system during pain induced stress.

Most of the subjects have been working in the factory for several years; with venepuncture being part of the six-monthly medical examinations. Most of the subjects indicated that venepuncture was not a stressful procedure to them. However, there was still a positive increase in SAA (percentage change of SAA compared to T1) on the venepuncture day. In contrast, the SAA levels were remained constant throughout the four-time points on the control day.

In another study (9), 24 healthy adults were exposed to the TSST – a standardized psychosocial stress test and a control (rest) condition on separate days. SAA levels showed a marked increase during the stress day but showed no significant changes during the control day. This showed that a psychological stressor can also increase SAA levels.

In the subjects undergoing venepuncture during a periodic medical examination, it is probable that both psychological as well as physical stressors contribute to the increase in SAA.

During the venepuncture, the increase in SAA from baseline is higher among those who reported greater self-perceived stress during the procedure. This is indicated in the positive trend in the difference of SAA between T3 and T1 among subjects with increasing PSL scores. T3–T1 values were 0.6 (among low PSL group *n* = 24), 11.3 (among moderate PSL group *n* = 18), and 78.9 (among high PSL group *n* = 16). This suggests that perceived stress can modify the change in SAA.

Furthermore, similar fluctuations in SAA were not seen on the control day over the same 30-min period. Although the baseline values of SAA were higher on the control day, SAA relative increment over four-time points was significantly higher on the venepuncture compared to the control day (*P* = 0.021). This suggests that venepuncture does cause a real increase in SAA.

There were some limitations of the study. The saliva samples were collected at 15-min (T1) and 1-min (T2) before venepuncture/control day activities, and 1-min (T3) and 15-min (T4) after that. Considering that the levels of SAA were still elevated after 15 min after venepuncture, additional time points for observation beyond 15 min should have been included in the study design. However, the workers had to go back to work after the medical examination, and the management was not willing to allow them to spend extra time for the study. Besides age, gender, and psychiatric illness, which were considered in this study, other covariates e.g., personality or individual psychological makeup, might confound the results of this study and need to be considered.

The sample size was not calculated prior to this study, as the maximum study size possible, comprising the entire lead exposed male population of the factory was studied.

## Conclusion

Salivary α-amylase increases in response to the acute stress of venepuncture during a periodic medical examination, and remains elevated 15 min after the procedure. Such fluctuations were not seen on a control day. During venepuncture, increase in SAA from baseline is higher among those who reported greater self-perceived stress during the procedure.

Further research is required in this area, e.g., studies with larger sample sizes and which include additional observations for time points beyond 15 min after the venepuncture, and to examine possible interactions between psychological and physical stressors on SAA levels.

## Conflict of Interest Statement

The authors declare that the research was conducted in the absence of any commercial or financial relationships that could be construed as a potential conflict of interest.
